# Fecal microbiota transplantation: a novel strategy for treating Alzheimer’s disease

**DOI:** 10.3389/fmicb.2023.1281233

**Published:** 2023-11-16

**Authors:** Wu Xiang, Han Xiang, Junyu Wang, Yiqin Jiang, Chuanhui Pan, Bingjin Ji, Anren Zhang

**Affiliations:** ^1^Department of Rehabilitation, Beibei Traditional Chinese Medical Hospital, Chongqing, China; ^2^Department of Rehabilitation Medicine, Shanghai Fourth People’s Hospital Affiliated to Tongji University School of Medicine, Shanghai, China; ^3^Department of Radiology, Daping Hospital, Army Medical University, Chongqing, China

**Keywords:** fecal microbiota transplantation, gut microbiota, Alzheimer’s disease, review, mechanism

## Abstract

Alzheimer’s disease is a common neurological disorder, which has become one of the major factors affecting human health due to its serious impact on individuals, families and society. It has been confirmed that gut microbiota can affect the occurrence and development of Alzheimer’s disease. Especially, fecal microbiota transplantation plays a positive role in the treatment of Alzheimer’s disease. The mechanisms for improving Alzheimer’s disease might include anti-inflammation and regulation of amyloid β-protein, synaptic plasticity, short-chain fatty acids, and histone acetylation. In this mini-review, the relationship between fecal microbiota transplantation and Alzheimer’s disease was summarized. It is hoped that fecal microbiota transplantation would play a positive role in the prevention and treatment of Alzheimer’s disease in the future.

## 1. Introduction

Alzheimer’s disease (AD) is a common neurodegenerative disease and the most common cause of dementia. The pathological features of AD mainly include deposition of amyloid β-protein (Aβ) (Duyckaerts et al., 2009; Bloom, 2014; Jouanne et al., 2017), neurofibrillary tangles caused by hyperphosphorylated tau protein (Duyckaerts et al., 2009; Bloom, 2014), and enhancement of neuroinflammation (Felsky et al., 2019). AD is usually characterized by cognitive deficits, as well as impairments in expressive speech, visuospatial processing, and executive function (Knopman et al., 2021). Epidemiology studies show that over 50 million people worldwide have been affected by AD (Hodson, 2018). According to the World Alzheimer’s Disease Report, the number of people with dementia is expected to increase to 139 million by 2050 due to an aging population, among which AD accounting for approximately 60–80% (World Alzheimer Report, 2022).

The etiology of AD may be the result of the interaction between multiple factors, including age, genetic factors, family history, lifestyle actors, and environmental factors. For example, the percentage of AD patients increases dramatically with age. It has been found that 5% of people age 65 to 74, 13.1% of people age 75 to 84 and 33.3% of people age 85 and older suffer from AD (Rajan et al., 2021; Wang et al., 2021). Meanwhile, many genes can increase the risk of AD, among which apolipoprotein E4 is the greatest effect on risk of late-onset AD (Bellenguez et al., 2022). People who have or had a parent or sibling with AD are more likely to develop the disorder (Loy et al., 2014). Genetic and non-genetic factors (e.g., diet and exercise) may play a role when disorders spread within families (Alzheimer’s Association, 2023). In addition, gender (World Alzheimer Report, 2022), smoking (Jeong et al., 2023), education (Manly et al., 2022), traumatic head injury (Schneider et al., 2021), cardiovascular disease (Samieri et al., 2018), gut microbiota (Kim et al., 2020), and other heterogeneous factors (Abolhasani et al., 2023) have been reported to be related to AD. These studies show that AD poses an enormous threat to global health because of high incidence rate, lacking effective efficacious pharmacotherapy and poor prognosis. Therefore, it is necessary to develop new therapeutic approaches to solve this disease.

There are about 100 trillion microorganisms in intestine (Valdes et al., 2018), which play an important role in maintaining the balance of the human body. At the same time, due to their key role in the regulation of the central nervous system, the gut microbiota is named the human “second brain” (Ridaura and Belkaid, 2015). There is a gut-brain axis between gut microbiota and the central nervous system (Morais et al., 2021; Mayer et al., 2022). The gut-brain axis consists of bidirectional communication between the enteric nervous system and the central nervous system, including the immune system, tryptophan metabolism, the vagus nerve and the enteric nervous system (Cryan et al., 2019). Relevant studies have confirmed that gut microbiota can slow down the AD progression by regulating brain function (Dodiya et al., 2019; Elangovan et al., 2019; Fujii et al., 2019; Zhou et al., 2019; Hazan, 2020; Park et al., 2021; Jin et al., 2023).

The gut microbiota of patients with AD is disordered. Research shows that the diversity of α and β of gut microbiota was reduced in patients with AD compared to healthy people (Liu et al., 2019). The gut microbiota disturbance has also been demonstrated in animal models (Harach et al., 2017). Currently, it has become a new strategy in the treatment of AD by targeting regulation of gut microbiota. Prebiotics (Sun et al., 2019a), probiotics (Akbari et al., 2016), and antibiotics (Angelucci et al., 2019) are associated with improvement of prognosis in AD. However, fecal microbiota transplantation (FMT) may be more effective, which involve a broader range of microbiome modifications than prebiotics, probiotics, or antibiotics (Smits et al., 2013). It has been shown that the symptoms of AD can be improved by FMT. Compared with mice from the same background and conventional breeding, FMT can regulate the disturbance of gut microbiota and improve their cognitive function in AD mice (Yang, 2018; Sun et al., 2019b).

In this mini review, the current knowledge about the relationship between gut microbiota and AD has been introduced, including the relationship between AD and gut microbiota, as well as the possible mechanisms of the effects of FMT. In addition, the research progress in FMT for AD has also been summarized (Table 1). Finally, we emphasize that FMT may become a new therapy for the treatment of AD.

**TABLE 1:** Summary of the application of FMT in the intervention of AD.

References	Experimental model	FMT intervention	Duration of intervention	Results
Dodiya et al., 2019	Recipients: 7-week-old antibiotic-treated APPPS1-21 male mice (*n* = 9); donors: age-matched APP PS1-21 male mice (*n* = 9)	Gastric gavage	Once a day until sacrifice	Reduced cecum weight, increased abundance of Bacteroides, Prevotella, and S24-7, resulted in the restoration of diversity and restored Aβ amyloidosis and microglia morphology
Elangovan et al., 2019	Tg-FO: 32-week-old 5xFAD mice, age-matched WT donors (*n* = 8); Tg-FY: 32-week-old 5xFAD mice, 10-week-old WT donors (*n* = 8)	Oral gavage	7 consecutive days	Improved spatial and recognition memory, enhanced recognition memory and reduced cortical Aβ loading.
Fujii et al., 2019	Recipients: 4-week-old germ-free C57BL/6N mice (*n* = 7); donors: AD patient and age-matched healthy volunteers (*n* = 7)	Infusion	75 weeks	Reduced the levels of GABA, taurine, tryptophan, tyrosine, and valine, increased propionate, worsened cognitive function, reduced abundance of Verrucomicrobia and Proteobacteria
Sun et al., 2019b	Recipients: APP/PS1 mice (*n* = 8); donors: WT mice (*n* = 8)	Oral gavage	Once a day for 4 weeks	Reduced levels of Aβ, COX-2, CD11b, Aβ40, Aβ42 and phosphorylation of tau protein; increased PSD-95 and synapsin I expression, reversed the changes of gut microbiota and SCFAs.
Zhou et al., 2019	Recipients: male 8-week-old SD rats injected with aggregated Aβ_1–42_ (*n* = 6); donors: same as recipients, treated with xanthoceraside or sham (*n* = 6)	Oral gavage	Once a day for 16 days	Reversed gut microbiota dysbiosis in AD animals, changed the abundances of several phyla and genus of bacterial.
Wang et al., 2020	Recipients: rats were treated with MnCl2 (*n* = 10); donors: SPF Sprague–Dawley male rats (*n* = 6)	Oral gavage	5 weeks	Reduced Aβ and Tau expression, downregulated NLRP3 and neuroinflammatory factors expression in the cerebral
Kim et al., 2020	Recipients: ADLR^APT^ mice (*n* = 12); donors: WT mice (*n* = 12).	Oral gavage	Once a day for 16 weeks	Ameliorated the formation of Aβ plaques and neurofibrillary tangles, glial reactivity and cognitive impairment, reversed abnormalities genes expression.
Hazan, 2020	Recipients: an 82-year-old male with CDI and 5-year history of AD; donors: the patient’s 85-year-old wife	Infusion	Single infusion	Improved mental acuity and affect, memory and mood, increased the MMSE scores.
Li et al., 2020	Recipients: young (∼ 3 months) male SD rats; donors: aged (20 ∼ 24 months) male SD rats	Gavage	once a day for 3 days, then twice a week for 2 months	Impaired cognitive behavior, changed synaptic structures and decreased dendritic spines, reduced BDNF, NMDA receptor NR1 subunit and synaptophysin expression, increased AGEs, RAGE, pro-inflammatory cytokines and oxidative stress expression.
Shen et al., 2020	FMT-AD: APP/PS1 mice with AD human donor (*n* = 5); FMT-AD-HP: APP/PS1 mice with healthy human donor (*n* = 5); Con-FMT-AD: WT mice with AD human donor (*n* = 5).	Oral gavage	28 days	FMT-AD: increased the expression of NLRP3, inflammatory factors, activated microglia. FMT-AD-HP: reduced the expression of NLRP3 and neuroinflammatory factors, suppressed the activation of microglia. Con-FMT-AD: increased the expression of NLRP3 and inflammatory factors.
D’Amato et al., 2020	Recipients: antibiotic treatment male C57BL/6 mice (*n* = 12); donors: adult (3 months) and aged (24 months) male C57BL/6 mice (*n* = 12)	Oral gavage	6 days	Impaired spatial learning and memory, reduced bacteria associated with SCFAs and CNS disorders, and acquired an ageing-like phenotype of microglia cell.
Kim et al., 2021	Recipients: 8-week-old C57-BL/6 (*n* = 8); donors: 9-month-old WT and 5xFAD mice (*n* = 8)	Oral gavage	5 consecutive days	Reduced the expression of adult hippocampal neurogenesis and brain-derived neurotrophic factor, increased p21 expression and pro-inflammatory cytokines.
Wang et al., 2021	Recipients: 3-month-old SPF APP/PS1 mice (*n* = 4); donors: 16-month-old APP/PS1 mice (*n* = 4).	Oral gavage	7 consecutive days	Increased Aβ plaques, suppressed astrocyte activation
Park et al., 2021	Recipients: 90-year-old woman with CDI, 5-year history of AD; donors: a 27-year-old man with healthy	Colonoscopy	2 infusions 3 months apart	Improved cognitive function, changed the microbiota composition and short-chain fatty acids.
Zhang et al., 2022	Recipients: 8-month-old adult male C57BL/6N mice; donors: WT mice.	None	None	Improved the gut permeability, reduced the levels of fecal LCN2, lowered the levels of serum pro-inflammatory factors TNF-α and MCP-1, reversed the microglia activation and Aβ protein deposition.
Hang et al., 2022	Recipients: Tg mice (*n* = 6); donors: tumor-bearing mice or WT mice (*n* = 6)	Gavage	3 times a week for 1 month	Improved short-term memory level and cognitive ability, regulated Inflammatory factors in the plasma, decreased A plaques burden in the hippocampus and cortex, and reversed the metabolism of inorganic and organic salts in the intestinal flora.
Jin et al., 2023	Recipients: newly weaned WT mice (*n* = 8); donors: 12-month-old APP/PS1 mice (*n* = 8)	Gavage	3 times a week for 1 month	Increased gut BACE1 and Aβ42 levels.

SOD, superoxide dismutase; BDNF, brain derived neurotrophic factor; NT3, neurotrophin-3; NGF, nerve growth factor; GABA, γ-aminobutyric acid; MMSE, mini mental status examination; NMDA, *N*-methyl-D-aspartate; CNS, central nervous system; AGE, advanced glycation end products; RAGE, receptor for advanced glycation end products.

## 2. Alzheimer’s disease and gut microbiota

As the largest microbiota in the human body, the human gut microbiota has over 1000 species. “Healthy microbiome” is defined as an ideal set of a healthy “functional core”: a complement of metabolic and other molecular functions that are performed by the microbiome within a particular habitat (Shafquat et al., 2014). Such a core might need to be present as genetic potential, and it must include at least the housekeeping functions necessary for individual microbial life (Lloyd-Price et al., 2016). Meanwhile, a healthy microbiome may be characterized further by the resistance of a microbiome to stress and perturbation and its ability to recover to a healthy functional profile afterward (Bodelier, 2011; Backhed et al., 2012).

Several studies have shown that the gut microbiota of AD patients or model animals is disrupted. For example, compared with age matched wild-type mice, the abundance of Odoribacter and Helicobacter increased in AD mice, while Prevotella decreased (Shen et al., 2017). Another study found that the abundance of Verrucomicrobia and Proteobacteria increased in AD mice, while Ruminococcus and Butyricicoccus decreased (Zhang et al., 2017). In clinical studies, the abundance of Firmicutes and Actinobacteria decreased in AD patients, while Bacteroidetes increased. Meanwhile, the phylum with elevated abundance was negatively correlated with Aβ1-42/Aβ1-40, and positively correlated with P-tau and P-tau/Aβ1-42 (Vogt et al., 2017).

Previous studies have found that transplanting gut microbiota of AD patients or mice into germ-free mice can lead to significant cognitive deficits in object localization and recognition. In contrast, FMT of healthy patients or mice can reverse symptoms and pathologic manifestations in AD model animals. For example, it has been demonstrated that cognitive deficits caused by deposition of Aβ and neurofibrillary tangles can be improved by FMT from healthy mice donors (Kim et al., 2020). Meanwhile, FMT could improve cognitive function by reducing deposition of Aβ, attenuating glial cell hyperactivation and secondary neuroinflammation, and decreasing blood-brain barrier (BBB) permeability (Sun et al., 2019b; Kim et al., 2021). The above evidence suggest that gut microbiota is closely related to the occurrence and development of AD. Based on the existing evidence, it is suggested that improving cognitive function by regulating gut microbiota may provide new ideas for the prevention and treatment of AD.

## 3. Definition and process of FMT

Fecal microbiota transplantation is a technology that places stool from a healthy donor into another patient’s gastrointestinal tract to change the recipient’s gut microbiota, thereby gaining therapeutic benefits (Gupta and Khanna, 2017). The first records of FMT have been traced back to the fourth century China, where it was used in patients with severe diarrhea (Zhang et al., 2012). Studies have shown that FMT has a positive effect on various diseases, including Clostridium difficile infection (CDI) (Hamilton et al., 2012), inflammatory bowel disease (Paramsothy et al., 2017), metabolic syndrome (Mocanu et al., 2021), autoimmune disorders (Yang et al., 2023), and neurological disorders (Xu et al., 2015). It is worth noting that FMT is also beneficial for AD (Yang, 2018; Sun et al., 2019b).

The process of FMT is rigorous. First of all, strict donor screening tests of FMT are needed (Cammarota et al., 2017), including a donor questionnaire, additional interview, standard donor screening protocols and the time between screening and donation. Secondly, patients undergoing FMT need support and education prior to treatment and antibiotics are avoided 12–48 h before fecal infusion (Blackburn et al., 2015). Finally, the current administration of stool by means include oral capsule, lower gastrointestinal route (via colonoscopy or retention enema) and upper gastrointestinal route (via nasogastric, nasojejunal, esophagogastroduodenoscopy, or nasoduodenal tube) (Wang et al., 2019). For example, a study showed that a non-significant difference in cure rate of recurrent CDI between upper and lower gastrointestinal routes of FMT (Youngster et al., 2014). However, another study found that the lower gastrointestinal route had higher clinical cure rate than the upper gastrointestinal route in CDI patients (Kassam et al., 2013). Furthermore, FMT via oral capsules had comparable results to delivery by colonoscopy in prevention of recurrent CDI (Kao et al., 2017). To sum up, there is no current strong evidence of the optimal FMT measure has been proved in clinical treatment, and it is recommended to select according to individual situation of patients.

## 4. Biological mechanisms of FMT to improve AD

More and more evidence showed that FMT may have potential for the prevention and treatment of AD. A randomized controlled trial suggested that the abundance of Proteobacteria and Verrucomicrobia decreased, while Bacteroidetes increased, in the FMT-treated mice. Meanwhile, the spatial learning ability and familiarity with novelty performed of FMT-treated mice better than AD model mice (Sun et al., 2019b). A randomized controlled trial suggested that the abundance of Proteobacteria and Verrucomicrobia decreased, while Bacteroidetes increased, in the FMT-treated mice. Meanwhile, the spatial learning ability and familiarity with novelty performed better than AD model mice (Sun et al., 2019b). Another study showed that FMT could reverse the disturbance of gut microbiota in AD mice and enhance the learning and memory ability (Yang, 2018). Numerous studies have shown that FMT can improve AD, which may be related to reducing the abundance of pathogenic bacteria, exerting the anti-inflammatory effects, decreasing the deposition of Aβ, regulating synaptic plasticity, increasing short chain fatty acids, and curbing histone acetylation.

### 4.1. Anti-inflammatory mechanisms

Inflammation plays an important role in the occurrence and development of many diseases, including metabolic disorders, immune system disorders, cardiovascular diseases and nervous system diseases.

The high expression of inflammatory factors can lead to metabolic disorders of neurotransmitters and disrupt the regulation and signaling mechanisms of behavior and cognition (Johnson et al., 2021). Research have found that the level of interleukin-1β (IL-1β), interleukin-6 (IL-6) and tumor necrosis factor-α (TNF-α) is significantly increased in the cerebrospinal fluid and peripheral blood of AD patients (Cattaneo et al., 2017). The symptoms of AD could be improved by inhibition of pro-inflammatory cytokines and enhancement of anti-inflammatory cytokines. For example, the defect of exercise behavior induced by lipopolysaccharide improved and the number of activated microglia of nigra reduced while the IL-1 receptor antagonists was added to the brain of P70 newborn rats (Pang et al., 2015). Meanwhile, the cognitive and memory function of transgenic male mice with IL-6 knock-out was lower than wild-type control mice (Hryniewicz et al., 2007). A 6-month prospective, single-center, open study showed that the cognitive function of AD patients was improved by TNF-α inhibitor enalapril (Tobinick et al., 2006). In addition, FMT could increase the level of anti-inflammatory cytokines, such as interleukin-10 (IL-10) and interleukin-22 (IL-22) (Kim et al., 2021).

Fecal microbiota transplantation can inhibit the level of pro-inflammatory cytokines and increase anti-inflammatory cytokines. For example, a study showed that manganese could induce the deposition of Aβ and production of tau protein, increasing inflammatory cytokines (e.g., IL-1β) and NLRP3 inflammasomes. FMT from healthy rat donors relieve neurotoxicity by reversing the above changes, but the composition of FMT is unclear (Wang et al., 2020). Another study found that FMT from AD mice donors led to impaired cognitive impairment, activated microglia, and increased NLRP3 and inflammatory factors (including IL-1β, IL-18, and TNF-α). The above changes could be reversed by FMT from healthy people donors, but the composition of FMT is unclear (Shen et al., 2020). In addition, the level of pro-inflammatory cytokines increased, such as TNF-α and monocyte chemoattractant protein-1 (MCP-1), in AD mice. Pearson correlation analysis showed that Ruminiclostridium_5 was positively correlated with inflammatory markers TNF-α and MCP-1. Nevertheless, FMT from wild type mice significantly decreased serum TNF-α and MCP-1 levels, which may be associated with reducing the abundance of Ruminiclostridium_5 (Zhang et al., 2022). Hang et al. (2022) showed that FMT treatment could increase the level of anti-inflammatory factors IL-2 and transforming growth factor-β (TGF-β) and reduce pro-inflammatory factors TNF-α and IL-1β in AD mice. The results may be associated with the increased Firmicutes and Prevotella and the decreased Bacteroidetes, Bacteroides and Sutterella. Yang (2018) found that the level of anti-inflammatory factors increased, such as IL-10, and the pro-inflammatory cytokines decreased, such as TNFα and IL-6, in the FMT-treated mice. The results may be associated with the increased Firmicutes, Bacteroidales, Verrucomicrobia, Clostridia, and Bacteroidia, and the decreased Erysipelotrichia, Clostridia, and Bacteroidia. Xu et al. (2020) showed that yeast β-glucans could increase beneficial bacteria and reduce pathogenic bacteria in the gut microbiota of AD mice, and reverse the increased IL-1β, IL-5, IL-6, and INF- γ and the decreased IL-10. Meanwhile, spearman correlation analyses showed that there were three negatively correlated (Oscillibacter, Butyricicoccus, and Mucispirillum) and two positively correlated (Lactobacillus and Bifidobacterium) with anti-inflammatory. Inversely, there were two negatively correlated (Lactobacillus and Bifidobacterium) and six positively correlated (Alistipes, Oscillibacter, Butyricicoccus, Rikenella, Mucispirillum, and Anaerotruncus) with pro-inflammatory. The above studies suggest that FMT may have a positive effect on cognitive function by regulating the gut microbiota, reducing the expression of pro-inflammatory cytokines, and increasing anti-inflammatory factors.

### 4.2. Regulating Aβ protein and synaptic plasticity

Previous studies have shown that excessive deposition of Aβ can accelerate the cascade of oxidative stress and neuroinflammation, and induce apoptosis of nerve cells. It has been reported that Aβ42 aggregation is the main cause of Aβ42 toxic protein, and the latter is the major reason of AD. The development of AD can be effectively prevented by inhibiting Aβ42 aggregation (Lansbury and Lashuel, 2006). Meanwhile, high level of Aβ was detected throughout the intestines of both mice and humans. The expression of Aβ42 increased in colons of mice after receiving the gut microbiota of aged APP/PS1 mice donors (Jin et al., 2023). Moreover, low density lipoprotein receptor-related protein 1 (LRP-1) overexpressing expression mice had reduced the level of Aβ in the blood and brain, which was associated with spatial learning, memory consolidation, and spatial recognition memory (Cheng et al., 2023). The above studies suggest a correlation between Aβ42 and AD.

It has been demonstrated that Aβ targets the modulation of synapses (Almeida et al., 2005), and impairment of synaptic plasticity is associated with cognitive decline in AD (D’Amato et al., 2020).

The definition of synaptic plasticity is the ability of synapses to adjust their function or shift shape in response to changes of internal and external environment. The time-dependent appearance of neurofibrillary tangles after deposition of Aβ can alter synaptic function, affect synaptic plasticity and cause synaptic loss in AD patients. Meanwhile, the memory and synaptic plasticity were damaged by oxidative stress induced by the imbalance between antioxidants and free radicals (Sehar et al., 2022). The change of synaptic plasticity in AD patients is characterized by synaptic dysfunction and impairment of synaptic morphology and structure, which regulated by neurotransmitters, synaptic plasticity related proteins and signaling pathways.

In recent years, the main target of synaptic plasticity mechanism studies is synapse-associated proteins, including growth-associated protein-43, synaptophysin, and postsynaptic density protein-95 (PSD-95). The synaptic function is inhibited by reduction of synapse-associated proteins. The expression of synaptic protein and PSD-95 decreased in AD mice. For example, the expression of PSD-95 in Tg2576 APP mutant neurons was lower than wild-type neurons (Almeida et al., 2005). In contrast, the synaptic plasticity enhanced by increasing the expression of PSD-95, thereby improving the reduction of synapse associated protein. Research showed that FMT from aged donors led to changed expression of proteins involved in synaptic plasticity and neurotransmission in young adult recipients. Meanwhile, four genera (Prevotellaceae, Faecalibaculum, Lachnospiraceae, and Ruminococcaceae) were found to be significantly differentially abundant compared to adult mice. There were three (Faecalibaculum, Lachnospiraceae, and Ruminococcaceae) were significantly correlated with proteins implicated in mitochondrial energy metabolism and neurotransmitter transport (D’Amato et al., 2020). Furthermore, FMT from aged donors led to significant changes in synaptic structure of young rats, which may be associated with reducing the abundance of Bacteroidetes, Prevotella, Bacteroides, and Parabacteroides. However, FMT treatment could reduce the brain deposition of Aβ40 and Aβ42 and increase the expression of PSD-95 and synapsin I in AD model mice, which may be associated with increasing the abundance of Desulfovibrionaceae (Sun et al., 2019b). Therefore, we speculate that FMT may improve AD by reducing deposition of Aβ and inducing synapse recurrent (Li et al., 2020).

### 4.3. Regulating the short chain fatty acids and histone acetylation

Short chain fatty acids (SCFAs) are the main metabolites of the gut microbiota, including acetate, propionate, butyrate, pentanoate and Caproate. SCFAs can regulate human homeostasis and play critical roles in biological functions. Many studies have shown that SCFAs are closely related to the occurrence and development of AD. A study found that the acetate concentration decreased in the AD model drosophila induced by Aβ deposition (Kong et al., 2021). Meanwhile, the concentrations of propionate, butyrate, and isobutyric acid in AD model mice was lower than wild-type mice (Zheng et al., 2019). In clinical studies, the expression of SCFAs in AD patients was the lowest compared to healthy humans and patients with mild cognitive impairment (Wu et al., 2021). An increase in SCFAs can reduce deposition of Aβ and improve cognitive impairment in AD mice. For example, the reshaped gut microbiome and enhanced butyrate formation are highly associated with behavioral alteration and brain oxidative status, and SCFAs attenuated the behavioral disorders and Aβ accumulation in AD mice (Liu et al., 2021a). Furthermore, there was a strong correlation between the levels of increased SCFAs and reduced cognitive functions in AD mice (Liu et al., 2021b). These studies show that SCFAs play an important role in the occurrence and development of AD.

There is a closely relation between histone deacetylases (HDACs) and AD. In the hippocampus, HDAC1, HDAC2, and HDAC8 are reported to strengthen neuroinflammation associated with cognitive dysfunction, while HDAC3 decreases dendritic spine density and levels of proteins associated with synaptic plasticity (Yang et al., 2022). Meanwhile, it has been reported that the increased activity of HDAC6 related to memory impairment (Li et al., 2021). However, treatment with acetate was shown to reduce mRNA levels of HDAC2, HDAC5, HDAC7, and HDAC8 (Huang et al., 2021), which indicate that SCFAs can regulate the expression of HDACs. According to the report, SCFAs (such as butyrate) can inhibit the activity of histone deacetylase (Tan et al., 2014), reduce deposition of Aβ in neurons. Treatment with butyrate can improve cognitive functioning in AD mice by adding hippocampal acetylation and increasing the expression of genes associated with synaptic plasticity (Govindarajan et al., 2011). At the same time, butyrate can serve as a histone deacetylase inhibitor to enhance the acetylation of histones adjacent to the neurotrophic factor promoter, thereby improving memory (Barichello et al., 2015). Furthermore, acetyl CoA converted by acetate can serve as an acetyl donor for histone acetylation, thus altering the inflammatory signaling in the microglia of brains (Shi and Tu, 2015). The above studies suggest that SCFAs may improve AD by affecting histone acetylation.

Many studies showed that FMT treatment could improve AD by regulating gut microbiota and increasing the expression of SCFAs. For example, a study showed that FMT from aged donors led to impaired spatial learning and memory in young adult recipients, which may be associated with a strong reduction of bacteria associated with SCFAs production (Lachnospiraceae, Faecalibaculum, and Ruminococcaceae) (D’Amato et al., 2020). However, yeast β-glucans could reverse the increased Firmicutes, Oscillibacter, Mucispirillum, and Butyricicoccus, and the decreased Bacteroidetes, Lactobacillus, and Bifidobacterium in AD mice (Xu et al., 2020). Meanwhile, yeast β-glucans treatment was able to trigger the generation of SCFAs, which may be associated with increasing the abundance of Bacteroidetes, Lactobacillus and Bifidobacterium (Xu et al., 2020). Moreover, another study found that FMT could reverse the decreased Bacteroidetes in the Tg mice. Meanwhile, the level of butyrate was significantly increased in the FMT-treated mice, which may be associated with increasing the abundance of Bacteroidetes (Sun et al., 2019b). Therefore, we speculate that FMT may improve AD by increasing the inhibition of histone acetylation by SCFAs. Therefore, we speculate that FMT may improve AD by increasing the inhibition of histone acetylation by SCFAs.

## 5. Conclusion and future directions

Based on the above evidence, we conclude that FMT is beneficial for AD. AD has a serious impact on the health and quality of life of patients, and it is urgent to find low cost and few side effects intervention measures. However, there is relatively less research on the effects of FMT on AD, especially in clinical studies. So far, only three articles involved in FMT and AD, but no clinical trials have been published, among which one was terminated early due to the COVID-19 pandemic. Therefore, there is an urgent need for large-scale clinical trials to verify whether FMT can be used as a strategy for the treatment of AD. At the same time, seven gut microbiotas were defined as a good FMT, such as, Lachnospiraceae, Faecalibaculum, Ruminococcaceae, Bacteroidetes, Lactobacillus, Bifidobacterium, and Desulfovibrionaceae, which still needs to be further validation.

This mini-review summarized the evidence of FMT in the treatment of AD in recent years, which can bring significant benefits to the prevention and treatment of AD. FMT play a positive effect on AD through the anti-inflammatory effects, regulating deposition of Aβ, synaptic plasticity, SCFAs, and the histone acetylation. Our findings may provide evidence for fecal microbiota-related drug preparations as adjuvant therapy for the prevention and treatment of AD. In the future, the biological mechanism of improvement of AD of FMT still needs to be further clarified.

## Author contributions

WX: Writing – original draft, Writing – review and editing. HX: Writing – original draft, Writing – review and editing. JW: Writing – original draft, Writing – review and editing. YJ: Writing – review and editing. CP: Writing – review and editing. BJ: Conceptualization, Writing – review and editing. AZ: Conceptualization, Writing – review and editing.
